# Risk management of pregnant women and the associated low maternal mortality from 2008–2017 in China: a national longitude study

**DOI:** 10.1186/s12913-022-07721-z

**Published:** 2022-03-14

**Authors:** Jue Liu, Wenzhan Jing, Min Liu

**Affiliations:** 1 Department of Epidemiology and Biostatistics, School of Public Health, Peking University, Haidian District, No.38, Xueyuan Road, Beijing, 100191 China; 2 National Health Commission Key Laboratory of Reproductive Health, Peking University Health Science Center, No.38, Xueyuan Road, Haidian District, Beijing, 100191 China; 3 Peking University Institute for Global Health and Development, Peking University, No.5 Yiheyuan Road Haidian District, Beijing, 100871 People’s Republic of China

## Abstract

**Background:**

Reducing maternal mortality is one of the key targets of the Sustainable Development Goals (SDGs). In response to the impact of increased birth rate on maternal and child safety following the implementation of the two-child policy in 2013, the Chinese government implemented the risk management strategy (namely Five Strategies for Maternal and Newborn Safety, FSMNS) to reduce maternal mortality ratio (MMR). We aimed to analyze the changes in the proportion of pregnant women at high risk screened before and after the implementation of the risk management strategy and the association with maternal mortality during the two-child policy era in China.

**Methods:**

We conducted a nationwide longitudinal study using data obtained from the National Statistical Yearbook and the National Health Statistics Yearbook for all 31 provinces from 2008–2017 to assess and analyze the changes in the proportion of pregnant women at high risk screened before (2008–2013) and after (2014–2017) the implementation of the risk management strategy during the two-child policy era. We used generalized estimating equation (GEE) models to analyze the relationship between the proportion of pregnant women at high risk and MMR after controlling for sociodemographic factors, health resources, and other maternal healthcare factors.

**Results:**

In the past decade, the number of livebirths in China increased by 32.3%, from 13.3 million in 2008 to 17.6 million in 2017. The median proportion of pregnant women at high risk in 31 provinces increased by 64.8%, from 14.87% in 2008 to 24.50% in 2017. The annual rate of increase in the median proportion of pregnant women at high risk after the implementation of risk management (1.33%) was higher than that before the implementation (0.74%). The median MMR in China decreased by 39.6%, from 21.7 per 100,000 livebirths in 2008 to 13.1 per 100,000 livebirths in 2017. The univariate GEE models showed that MMR decreased by 7.9% per year from 2008–2017 (cRR 0.92, 95% CI 0.91–0.93), and the proportion of pregnant women at high risk was negatively correlated with MMR (cRR 0.97, 95%CI 0.94–0.99; p = 0.001). In the multivariate GEE models, after adjusting for confounders, the proportion of pregnant women at high risk remained negatively correlated with MMR. In the subgroup analysis, the association of MMR with GDP per capita and government health expenditure per capita existed only prior to the implementation of risk management; while high MMR was associated with a low proportion of pregnant women at high risk after the implementation of risk management.

**Conclusion:**

The national risk management strategy contributed to the stable decline of MMR in China during the two-child policy era. Further attention should be focused on pregnant women in China’s central and western regions to ensure reaching SDGs targets and the ‘Healthy China Plan’ by 2030.

**Supplementary Information:**

The online version contains supplementary material available at 10.1186/s12913-022-07721-z.

## Introduction

Maternal death is a major public health issue of common concern worldwide. It is estimated that 303 000 women died in 2015 during and following pregnancy and childbirth [1]. During the past three decades, although substantial progress has been made in maternal survival, regional inequalities persist [2]. 99% of all maternal deaths occur in developing countries and the majority could have been prevented [1, 2]. As a developing country with the largest population in the world, China is one of the few countries which has achieved the Millennium Development Goals (MDGs) to reduce the maternal mortality ratio (MMR) by three-quarters [3, 4], decreasing from 89 deaths per 100 000 livebirths in 1990 to 29 deaths per 100 000 livebirths in 2015 [5, 6]. The government’s strong commitment and implementation of strategies to ensure access to delivery care in health facilities showcase the remarkable progress in maternal survival in China during the MDGs era [7].

To expedite the decline, the Sustainable Development Goals (SDGs) and the Global Strategy for Women's, Children's and Adolescent's Health (2016–2030) set the target of reducing the global MMR to less than 70 deaths per 100 000 livebirths by 2030 [8, 9]. In October 2016, China committed to an important national medium and long-term strategic plan for the health sector to fulfill its international SDGs commitment, namely the Healthy China 2030 Plan, which was launched by the Central Committee of the Communist Party and the State Council in China [10, 11]. The target of MMR in the Healthy China 2030 Plan is to reduce the national MMR to 12.0 deaths per 100 000 livebirths by 2030 [12]. To achieve the targets in the SDGs and Healthy China 2030, sustained progress in reducing MMR needs to be achieved in China. However, as China relaxed its one-child policy in November 2013 [13], maternal and child health care challenges have arisen in the new multiparous era, such as the increased risk of potential complications of multiple and mature pregnancies, the shortage of the health workers required to meet the additional demand for maternal and newborn care services, and the increased demand for more sophisticated prenatal, peripartum and neonatal care [14].

To guarantee maternal and newborn safety in the new multiparous era, the National Health Commission developed the risk management strategy (named the Five Strategies for Maternal and Newborn Safety, FSMNS). All health facilities conducted dynamic screening, evaluation, grading and management of pregnancy-related risks for pregnant women from pregnancy to 42 days after the delivery date, to timely discover and control the risk factors of pregnancy, prevent adverse pregnancy outcomes, and ensure maternal and newborn safety. Risk screening for pregnant women is initiated during the first visit to a healthcare facility, and the secondary or tertiary healthcare facilities which have midwifery services conducted risk assessments and classified pregnancy risk into different levels. Medical records were then labelled with five colors of “green (low risk), yellow (general risk), orange (high risk), red (highest risk), and purple (infectious disease) to enhance classification management. High-risk mothers classified as orange, red, and purple were included for the case-by-case management as the key population during the perinatal period to ensure whole-process management, dynamic supervision, centralized treatment, and to further ensure the screening, registration, reporting, management, and treatment of each high-risk pregnant woman. By implementing the risk management strategy, China has unified the screening standards for high-risk pregnant women nationally, expanded the scope of high-risk pregnant women, and strengthened the management and referral of high-risk pregnant women. By improving the capability of maternal and child health workers in identifying high-risk pregnant women, more high-risk pregnant women were identified and managed at an early stage. The MMR in China is stable with a slight decline [15].

In the context of efforts to achieve universal health coverage, improving maternal health is critical to fulfilling the aspiration to reach SDG 3. The WHO has called for effective action and strategies to provide accessible, equal, proficient, and high-quality health care services to tackle the causes of maternal death globally [6]. However, knowledge about effective strategies to reduce MMR for developing countries is still lacking currently. Hence, this study will answer the following research question: What strategies can a country use to reduce the risk of death among pregnant women, specifically rapidly increasing numbers of pregnant women with limited health care resources? Has the risk management strategy contributed to the decline of MMR during the two-child policy era in China? In this study, we aimed to analyze the changes in the proportion of pregnant women at high risk screened before and after the implementation of the risk management strategy and the associated maternal mortality during the two-child policy era in China, to explore the factors that promote the reduction of maternal mortality, and to provide experience and evidence for other countries to develop appropriate strategies on reducing maternal mortality.

## Methods

### Study design

**Fig. 1:**
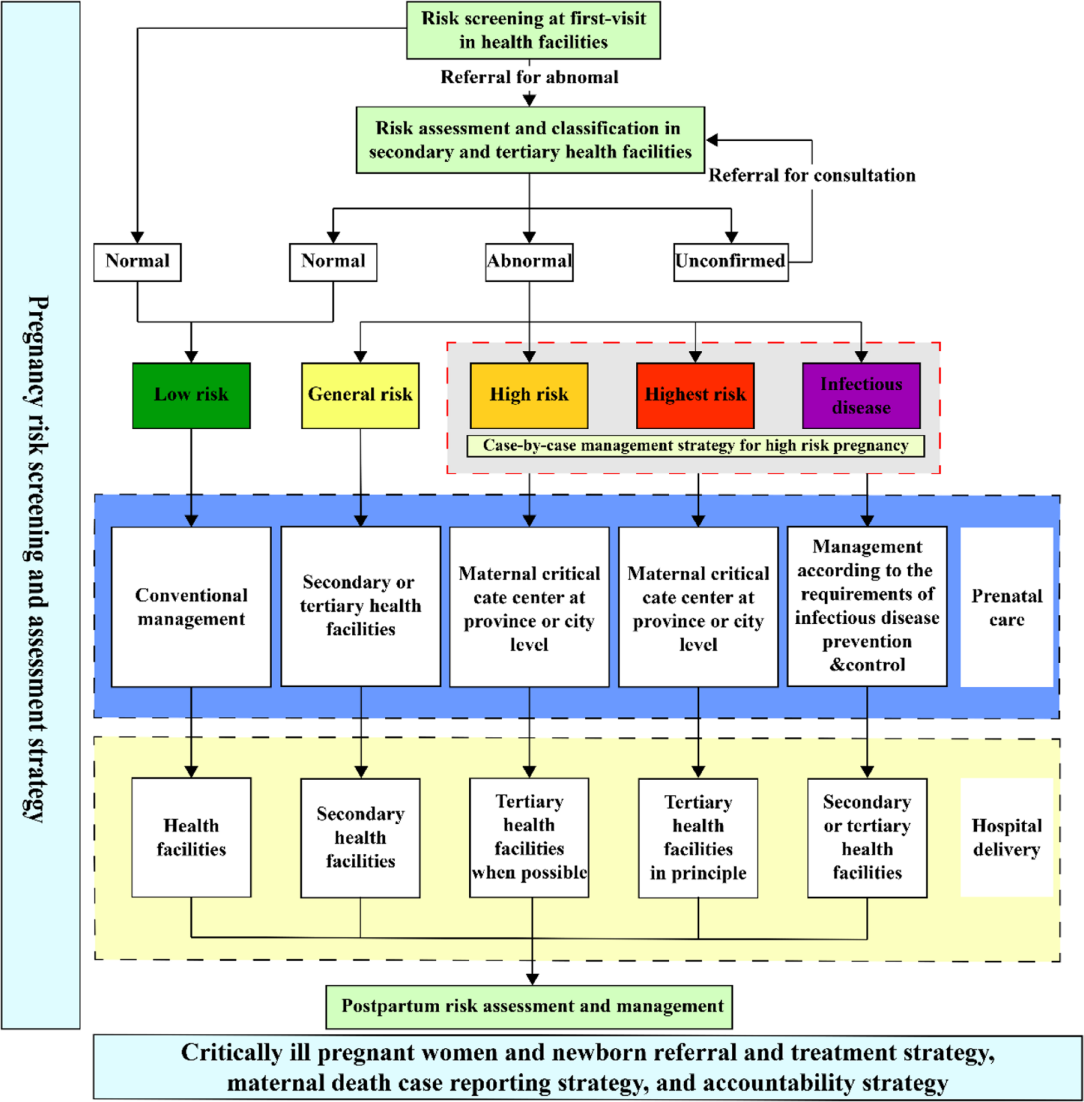
Risk management strategy among pregnant women in China

We conducted a nationwide longitudinal study to analyze changes in the proportion of pregnant women at high risk screened before the implementation of risk management (2008–2013) and after the implementation of risk management (2014–2017) in the past 10 years, and to analyze the associated maternal mortality after the adjustment of other confounding factors (i.e. sociodemographic status, medical resources, and maternal health care) that might affect the relationship of high-risk pregnant women and maternal mortality. After the adjustment of fertility policy in 2013, to guarantee maternal and newborn safety, the National Health Commission developed the risk management strategy (Supplemental Box 1), namely the Five Strategies for Maternal and Newborn Safety (FSMNS). The FSMNS strategy includes 1) a pregnancy risk screening and assessment strategy, 2) a case-by-case management strategy for high-risk pregnancy, 3) critically ill pregnant women and newborn referral and treatment strategy, 4) a maternal death case reporting strategy, and 5) an accountability strategy. Among them, the pregnancy risk screening and assessment strategy is the foundation for high-quality perinatal healthcare. Standardized trainings for the implementation of the FSMNS strategy have been conducted nationwide. All pregnant women are screened and assessed by medical workers using uniform standards. According to the level of risk, their medical records are labeled with green (low risk), yellow (general risk), orange (high risk), red (highest risk), or purple (infectious disease) for classification and risk management. High risk mothers (labelled with orange, red and purple) are included as a key population in the case-by-case management strategy for high-risk pregnancy during the perinatal period. Pregnant women with different risk levels are transferred to different level maternal and child health (MCH) institutions for perinatal care and delivery (Fig. 1). The government prioritizes limited high-quality medical resources for high-risk pregnant women to ensure maternal and newborn safety. The core concept of the risk management strategy to reduce the risk of maternal death is the screening and management of high-risk pregnant women, thus we use the proportion of pregnant women at high risk as a key management indicator in this study. The need for ethics approval was waived by the Institutional Review Board of Peking University. All methods were carried out in accordance with the declaration of Helsinki.

### Data sources

We extracted data of maternal health from the National Health Statistics Yearbooks [15], including the number of livebirths, maternal deaths, proportion of pregnant women at high risk, proportion of maternal systematic management, hospital delivery rate, and proportion of skilled birth attendance and sterile delivery in each province between 2008 and 2017. The data of maternal health in the National Health Statistics Yearbooks were from the National Maternal and Child Health Annual Reporting System. The National Maternal and Child Health Annual Reporting System was established during the early 1980s and covers the entire Chinese population with population-based data [16]. Since 1997, the National Maternal and Child Health Annual Report has established rigorous quality control mechanisms to ensure data reliability, including data audits, supervision, and standardization of data collection methods [7].

We obtained government health expenditures per capita, the number of licensed doctors per 1000 population, the number of licensed nurses per 1000 population, and number of beds in medical institutions per 1000 population in each province between 2008 and 2017 from the National Health Statistical Yearbooks in China [15]. In order to better analyze the association between the MCH-related resources and maternal mortality, we extracted the number of beds dedicated to gynecology, obstetrics, and pediatrics in medical institutions. Then we divided it by the number of livebirths to obtain the number of beds of gynecology, obstetrics, and pediatrics per 1000 livebirths in each province.

We also extracted sociodemographic data of 31 provinces from 2008 to 2017 from the National Statistical Yearbooks [17], including the resident population number, proportion of female illiterates aged 15 years or over, proportion of ethnic minorities, length of highways, crude birth rates, and gross domestic product (GDP) per capita in each province. In the National Statistical Yearbooks, the resident population number in 2010 is from the 2010 National Census [18] and the estimated number of resident population in the other years is based on the annual population sampling surveys.

### Proportion of pregnant women at high risk

The proportion of pregnant women at high risk is defined as the ratio of the number of high-risk pregnant women to the number of livebirths [15]. Among them, high-risk pregnant women refer to the number of women who have certain pathological factors during pregnancy that may harm pregnant women, fetuses, newborns or dystocia. The proportion of pregnant women at high risk in the 31 provinces between 2008 to 2017 was extracted from the National Health Statistics Yearbooks [15].

### Outcomes

MMR is the primary outcome in this study. We extracted the provincial data of MMR between 2008 to 2017 from the National Health Statistics Yearbooks [15], Provincial-level MMR in the National Health Statistics Yearbook are from the National Maternal and Child Health Annual Report. Maternal deaths are defined as deaths of women who are pregnant or death within 42 days of the termination of the pregnancy, irrespective of the duration and site of the pregnancy, or from any cause related to or aggravated by the pregnancy or management, but not from accidental or incidental causes, which is identical to the WHO's definition [19].

### Data analysis

MMRs were presented as median with interquartile range. We compared the proportion of pregnant women at high risk before the implementation of risk management (2008–2013) and after the implementation of risk management (2014–2017) in the past 10 years. We used heat maps to show the trends of the proportion of pregnant women at high risk and MMR at provincial level from 2008 to 2017 in China.

Generalized estimating equation (GEE) models were applied to estimate the association between proportion of pregnant women at high risk and maternal death, which dedicated for analysis of longitudinal data with repeated measures over time. We chose the most commonly used method (GEE) to examine the association of risk factors or policy-intervention factors on maternal mortality [7, 20, 21], and to make it comparable to previous studies. We used the GEE model with a negative binomial distribution and log link function to control for the skewed nature of maternal death. In the univariate GEE model, after controlling the effect of time, the association between the proportion of pregnant women at high risk and MMR was analyzed by including the yearly variables, the proportion of pregnant women at high risk and MMR in the model. Crude risk ratios (cRRs) with 95% confidence interval (CI) were calculated. In the multivariate GEE models, the association between the proportion of pregnant women at high risk and maternal death was examined after the adjustment of sociodemographic factors (including region, year, proportion of female illiterates aged 15 years and over, proportion of ethnic minorities, length of highways, crude birth rates, and GDP per capita), health resource (number of licensed doctors and nurses per 1000 population, number of beds of gynecology, obstetrics, and pediatrics per 1000 livebirths, and government health expenditures per capita), and other maternal health factors (proportion of maternal systematic management, hospital delivery rate, and proportion of skilled birth attendance and sterile delivery) by including all the above variables in the models. The adjusted risk ratios (aRRs) with 95% confidence interval (CI) were calculated. To examine the robustness of our findings, we performed a sensitivity analysis that adjusted only for maternal health factors and sociodemographic factors in the multivariate GEE models, instead of adjusting all the covariates in the full multivariate model. In the subgroup analysis, we assessed the relationship between the proportion of pregnant women at high risk and maternal death stratified by periods (before risk management and after risk management) after adjusting for other potential risk factors. All of the analyses were completed with SAS software, version 9.4 and Stata software, version 14. Two-sided p values of less than 0·05 were deemed to be statistically significant.

## Results

**Fig. 2:**
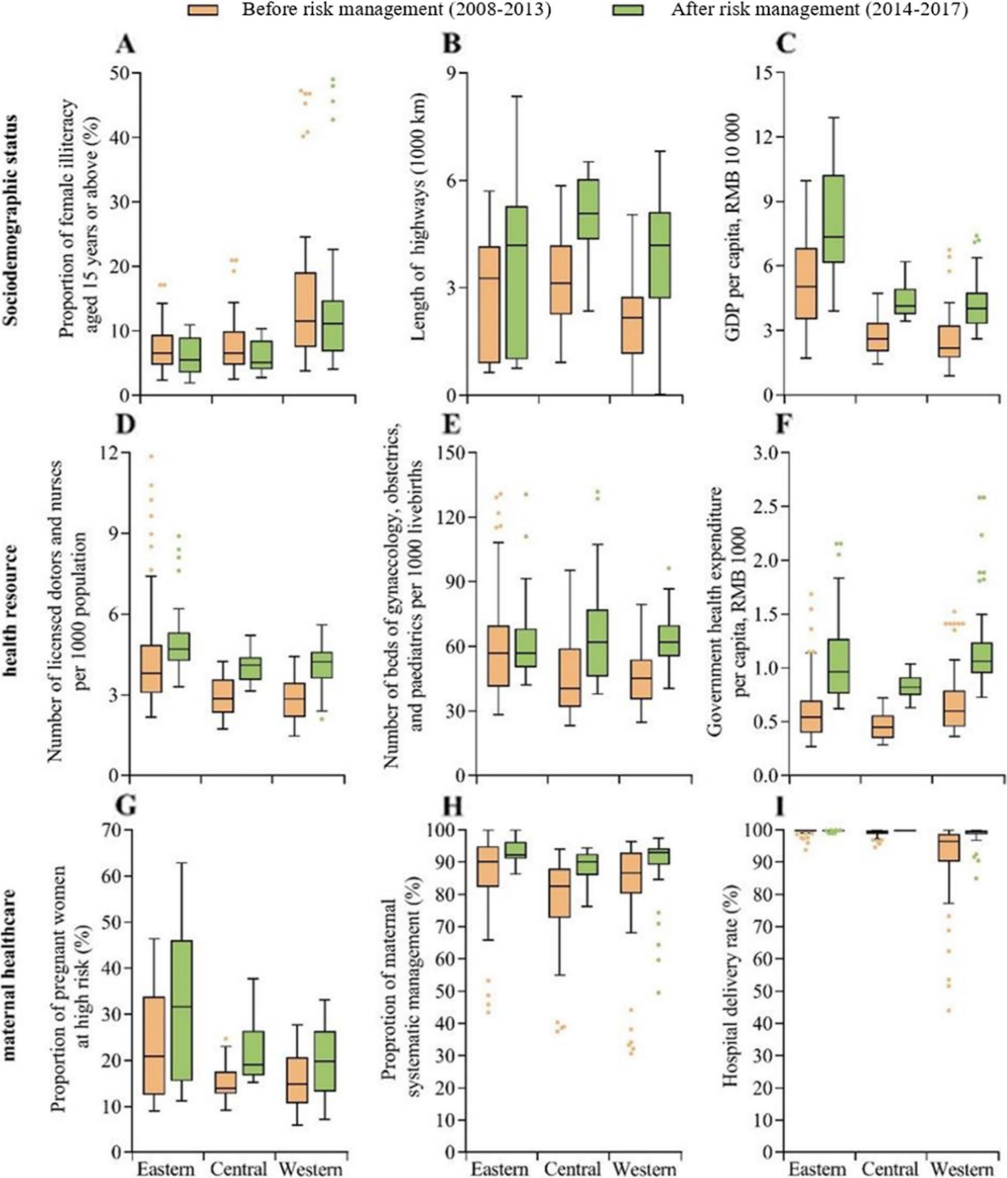
Sociodemographic status, health resource, and maternal healthcare parameters, by period

In the past decade, the number of livebirths in China increased by 32.3%, from 13.3 million in 2008 to 17.6 million in 2017. The median proportion of pregnant women at high risk in 31 provinces increased by 64.8%, from 14.87% in 2008 to 24.50% in 2017. The annual rate of increase in the median proportion of pregnant women at high risk after the implementation of risk management (1.33%) was higher than that before the implementation (0.74%). After the implementation of risk management, the median proportion of high-risk women screened in the eastern, central, and western regions was significantly higher than before the implementation (all P < 0.05, Table 1). There were also differences in sociodemographic factors, health resources, and other maternal healthcare factors in China during the two periods before and after the implementation of risk management (Fig. 2).

**Table 1:** Proportion of pregnant women at high risk before and after implementation of risk management

	Before risk management (2008–2013)		After risk management (2014–2017)	
	Livebirths (n)	Proportion of high-risk pregnant women (%)	Livebirths (n)	Proportion of high-risk pregnant women (%)
Total*	86,409,422	16.48 (12.10, 21.90)	65,768,781	22.45 (16.20, 30.85)
Region				
Eastern*	32,212,892	20.85 (12.63, 33.80)	27,045,494	32.60 (16.20, 47.85)
Central*	29,422,851	13.95 (12.85, 17.48)	20,381,043	19.10 (17.50, 27.15)
Western*	24,773,679	14.89 (10.70, 20.65)	18,342,244	19.85 (13.30, 27.25)

Data for proportion of pregnant women at high risk are showed as median (IQR). *P < 0.05

The median MMR in China decreased by 39.6%, from 21.7 per 100,000 livebirths in 2008 to 13.1 per 100,000 livebirths in 2017 (Table 2). The annualized rate of decline in MMR during 2008–2017 and the median MMR in 2017 in the western region was both higher than that in the eastern and central regions (Supplemental Fig. 1).

**Table 2:** Trends in maternal mortality in China from 2008 to 2017

Year	Number of livebirths	Maternal mortality ratio (per 100 000 livebirths)				
		Total		Eastern	Central	Western
		Median (IQR)	Range	Median (IQR)	Median (IQR)	Median (IQR)
Before risk management (2008–2013)						
2008	13,307,045	21.70 (15.95–35.20)	6.57–233.96	12.32 (7.79, 16.78)	21.36 (18.88, 28.06)	40.38 (28.09, 53.41)
2009	13,825,431	19.20 (13.70–30.10)	5.20–232.20	11.90 (9.50, 14.60)	18.10 (17.10, 22.50)	33.20 (26.30, 44.85)
2010	14,218,657	20.70 (12.10–29.70)	3.60–174.80	11.50 (7.40, 13.10)	18.55 (14.90, 24.30)	34.20 (22.90, 40.25)
2011	14,507,141	15.70 (10.20–22.80)	1.20–180.70	9.70 (6.40, 11.40)	16.10 (11.60, 16.70)	23.70 (20.15, 36.90)
2012	15,442,995	11.70 (9.90–24.40)	1.40–176.10	9.20 (4.00, 10.50)	11.60 (10.70, 16.90)	25.25 (18.15, 31.05)
2013	15,108,153	14.20 (10.20–17.90)	1.90–154.50	9.30 (8.30, 10.70)	14.25 (11.15, 15.80)	21.65 (15.30, 30.25)
After risk management (2014–2017)						
2014	15,178,881	14.10 (9.00–19.50)	1.90–108.90	8.80 (5.50, 10.30)	12.95 (10.10, 14.85)	19.55 (16.55, 30.05)
2015	14,544,524	11.10 (8.50–17.80)	2.30–101.00	8.30 (5.70, 8.50)	13.55 (9.80, 14.80)	19.40 (14.65, 27.75)
2016	18,466,561	12.70 (9.40–17.50)	2.20–109.90	9.20 (5.70, 10.50)	12.55 (9.65, 14.20)	18.75 (14.35, 27.40)
2017	17,578,815	13.10 (9.00–19.70)	1.10–95.00	8.30 (6.00, 10.40)	12.80 (10.00, 14.40)	17.35 (13.70, 26.45)

**Fig. 3:**
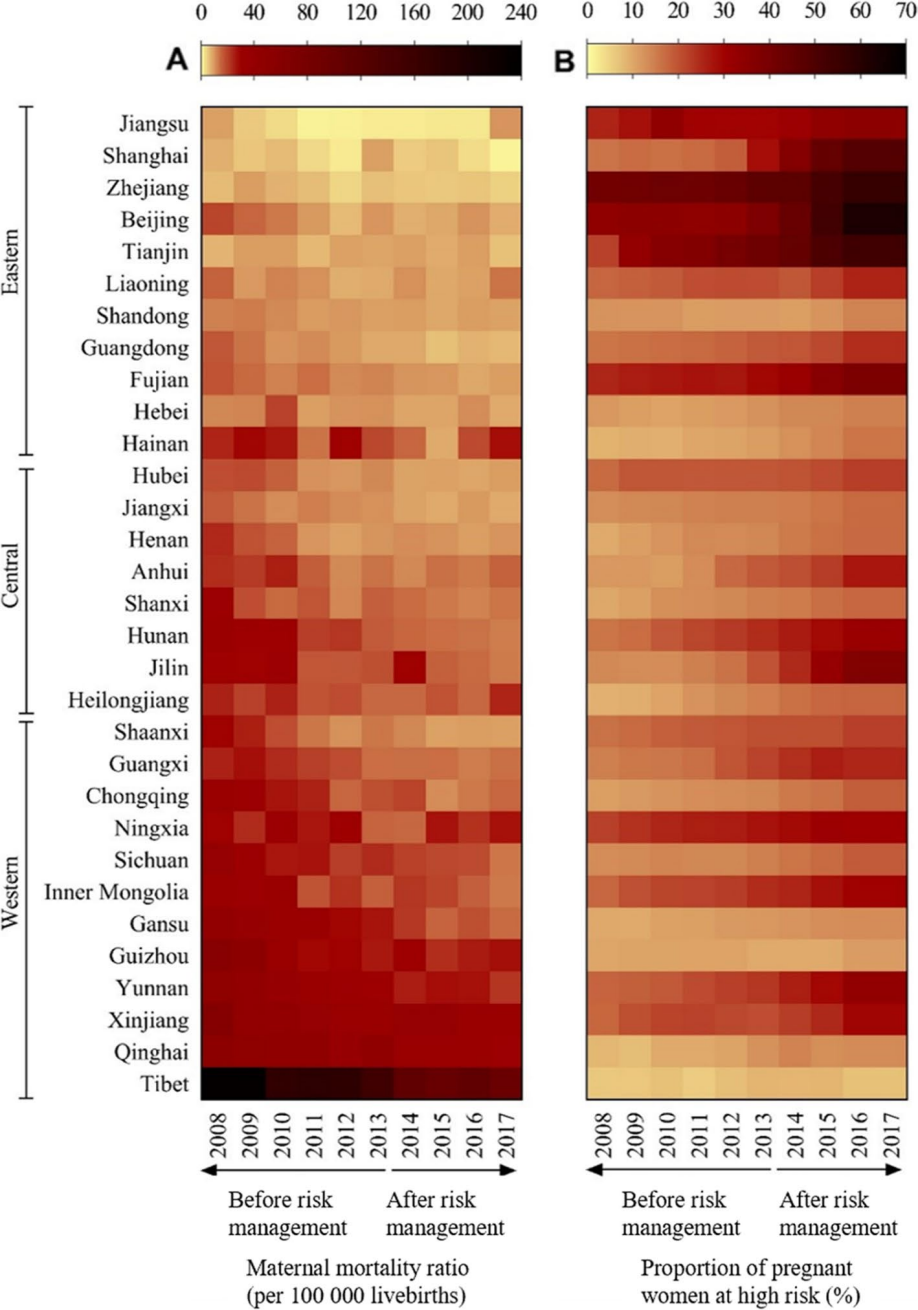
Trends of maternal mortality ratio and proportion of pregnant women at high risk in China

In the univariate GEE model, proportion of pregnant women at high risk was negatively correlated with MMR (RR 0.97, 95% CI 0.94–0.99, Fig. 3 and Table 3). In addition, region, year, proportion of ethnic minorities, length of highways, crude birth rates, and GDP per capita, and hospital delivery rates were also associated with MMR (Table 3). MMR decreased by 7.9% per year during 2008–2017 (RR 0.92, 95% CI 0.91–0.93), and MMR in the western region was significantly higher than that in the eastern region (RR 3.67, 95% CI 2.02–6.67).

**Table 3:** Association between the proportion of pregnant women at high risk and maternal mortality in the GEE model

Factors	Unadjusted model^a^		Adjusted model^b^	
	Risk ratio (95% CI)	*P* Value	Risk ratio (95% CI)	*P* Value
Proportion of pregnant women at high risk (%)	0.97 (0.94,0.99)	0.001*	0.99 (0.98,1.00)	0.002*
Region				
Eastern	1 (Reference)	-	1 (Reference)	-
Central	1.61 (1.22,2.13)	0.001*	1.33 (1.13,1.57)	0.001*
Western	3.67 (2.02,6.67)	< 0.001*	1.37 (1.14,1.65)	0.001*
Year	0.92 (0.91,0.93)	< 0.001*	0.97 (0.95,1.00)	0.035*
Proportion of female illiterates aged 15 years or over (%)	1.01 (0.99,1.03)	0.263	1.01 (1.00,1.02)	0.114
Proportion of ethnic minorities (%)	1.03 (1.02,1.03)	< 0.001*	1.01 (1.01,1.02)	< 0.001*
Length of highways (1000 km)	0.93 (0.90,0.96)	< 0.001*	0.94 (0.90,0.98)	0.005*
Crude birth rates (%)	1.03 (1.01,1.06)	0.016*	0.98 (0.95,1.01)	0.188
GDP per capita (10 000 RMB)	0.92 (0.87,0.97)	0.004*	0.91 (0.86,0.97)	0.004*
Number of licensed doctors and nurses per 1000 population	1.02 (1.00,1.05)	0.053	1.04 (0.98,1.09)	0.196
Number of beds of gynecology, obstetrics, and pediatrics per 1000 livebirths	1.00 (1.00,1.00)	0.674	1.00 (1.00,1.00)	0.791
Government health expenditures per capita (1000 RMB)	1.14 (0.83,1.57)	0.43	1.06 (0.84,1.34)	0.609
Proportion of maternal systematic management (%)	1.00 (0.99,1.01)	0.656	1.00 (0.99,1.00)	0.504
Hospital delivery rate (%)	0.98 (0.96,0.99)	0.008*	0.99 (0.98,1.00)	0.002*
Proportion of skilled birth attendance and sterile delivery (%)	0.99 (0.93,1.06)	0.734	1.00 (0.99,1.00)	0.733

^*^P < 0.05

^a^In the univariate GEE model, we controlled the effect of time

^b^Risk ratios were adjusted for sociodemographic factors (including region, year, proportion of female illiterates aged 15 years or over, proportion of ethnic minorities, length of highways, crude birth rates, and GDP per capita), health resource (number of licensed doctors and nurses per 1000 population, number of beds of gynecology, obstetrics, and pediatrics per 1000 livebirths, and government health expenditures per capita), and other maternal health factors (proportion of maternal systematic management, hospital delivery rate, and proportion of skilled birth attendance and sterile delivery)

In the multivariate GEE model, after adjusting for sociodemographic factors, health resources, and maternal healthcare factors, the proportion of pregnant women at high risk was still negatively correlated with MMR (aRR 0.99, 95% CI 0.98–1.00, Table 3). MMR decreased by 3% per year from 2008–2017 (aRR 0.97, 95% CI 0.95–1.00). In addition, western and central regions with a high proportion of ethnic minorities, inadequate length of highways, low GDP per capita, and low hospital delivery rates were associated with high MMR (p < 0.05). In the sensitivity analysis, the results were stable after adjusting only for maternal healthcare and sociodemographic covariates.

**Fig. 4:**
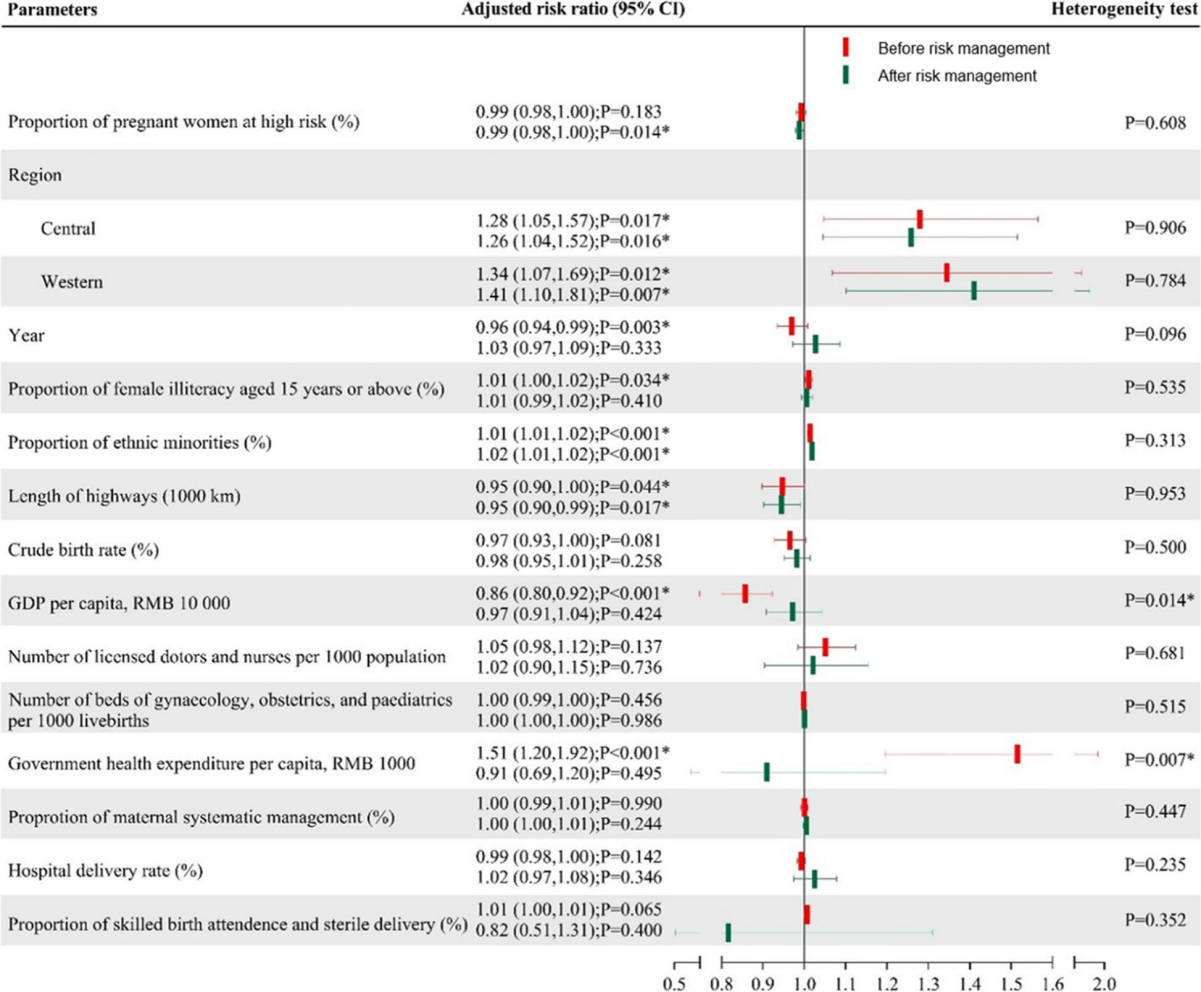
Association between the proportion of pregnant women at high risk and maternal mortality stratified by the implementation of risk management

In the subgroup analysis, association of MMR with GDP per capita and government health expenditure per capita existed only before the implementation of risk management; while high MMR was associated with a low proportion of pregnant women at high risk after the implementation of risk management (aRR 0.99, 95% CI 0.98–1.00, Fig. 4). Either before or after the implementation of risk management the western and central regions with ahigh proportion of ethnic minorities, inadequate length of highways were associated with high MMR (all P < 0.05, Fig. 4 and supplemental Table 1).

## Discussion

This is the first study that analyzed the changes in the proportion of pregnant women at high risk screened before and after the implementation of the risk management strategy and the associated maternal mortality during the two-child policy era in China. Our study found that the median proportion of pregnant women at high risk in 31 provinces in China increased by 64.8%, from 14.87% in 2008 to 24.50% in 2017, and the annualized rate of increase in the median proportion of pregnant women at high risk after the implementation of risk management was higher than that before the implementation. These might be related to the risk management based on FSMNS strategy adopted by the government after the implementation of the two-child policy, and might also be related to the increased proportion of pregnant women at high risk of comorbidities and complications. In November 2013, the Chinese government firstly loosened the one-child policy that had been implemented for three decades to the two-child policy. Previous studies showed that the proportions of pregnant women with advanced maternal age (over 40 years), multipara, irregular prenatal care, and referrals from lower-level hospitals were all significantly increased after the implementation of the two-child policy [22]. Moreover, prevalence of hypertensive disorder complicating pregnancy, gestational diabetes mellitus, placenta previa, placenta accrete spectrum, and severe postpartum hemorrhage was also increased significantly during the two-child policy era [22, 23]. The increased proportion of pregnant women at high risk of complications brought a heavier burden of critically ill pregnant women and also brought new challenges to the decrease of MMR during the two-child policy era [14, 24, 25]. Timely screening and effective management of pregnant women at high risk is crucial to reducing maternal mortality.

We found that the proportion of pregnant women at high risk was negatively correlated with MMR after controlling for other confounding factors. After the implementation of risk management, the proportion of high-risk pregnant women was negatively correlated with MMR, while there was no statistically significant association before risk management. These findings indicated that the risk management strategy promoted the MMR in China stably with a slight decline during the two-child policy era. Previous studies demonstrated that timely access to risk-appropriate obstetric and neonatal care could reduce perinatal mortality [26]. The risk management strategy ensures high-risk pregnant women timely receive appropriate obstetric care through risk screening, assessment, grading, and referral.

According to the FSMNS, risk screening is fundamental and crucial to identify pregnant women at relatively high risk of maternal death, which is a dynamic and ongoing strategy throughout the whole pregnancy cycle. For the high-risk mothers who are classified as orange, red, and purple in the management information system are included for the case-by-case management as the key population during the perinatal period to ensure whole-process management, dynamic supervision, centralized treatment, and to further ensure the screening, registration, reporting, management, and treatment of each pregnant woman at high risk. The yellow, orange, red, and purple classified pregnant women are transferred to secondary or tertiary health facilities to receive further maternal health services and hospital delivery. Pregnant women with different risk levels are transferred to different level maternal and child health institutions for perinatal care and delivery. The government prioritizes limited high-quality medical resources for high-risk pregnant women to ensure maternal and newborn safety. A nationwide network of critically ill pregnant women and newborn referral and treatment has been established, including a total of 3 369 maternal critical care centers and 3 070 neonatal critical care centers. Previous studies showed that the burden of maternal mortality has affected increasing numbers of women in some high-income countries [27, 28]. To reduce maternal mortality in the United States, a classification system for levels of maternal care was developed, including basic care (level I), specialty care (level II), subspecialty care (level III), and regional perinatal health care centers (level IV) [26]. Pregnant women at high risk could receive care in facilities that are prepared to provide the required level of specialized care, to better reduce maternal mortality in the United States [26].

MMR is one of the key indicators in the SDGs and Healthy China 2030. In this national longitudinal study, we found that MMR in 31 provinces decreased by 7.9% per year from 2008–2017. Our findings support that MMR has been significantly reduced due to risk management after the implementation of FSMNS. For example, the proportion of MMR caused by pregnancy-induced hypertension (the second leading cause of MMR in China) has reduced from 3.0 deaths per 100,000 livebirths in 2008 to 2.0 deaths per 100,000 livebirths in 2017, even though the number of pregnancy-induced hypertension has increased significantly after the two-child policy. Our findings also support that the overall MMR in China had reached the SDGs goal of reducing MMR to less than 70 deaths per 100,000 livebirths [8]. However, there is still a gap to reaching the goal of the Healthy China 2030 Plan which set the goal of reducing MMR to 12 deaths per 100,000 livebirths by 2030 [12]. Concerted efforts should be made to achieve the goal of Healthy China 2030 Plan on MMR in the future.

Moreover, we also found significant regional inequities exist in MMR. For example, MMR in Shanghai province from the eastern region was only 1.1 deaths per 100 000 livebirths in 2017, which is significantly lower than the MMR target set in Healthy China 2030 Plan and ahead of schedule. However, MMR in Tibet from the western region (95 deaths per 100 000 livebirths) is still far from the global level of SDG target. The regional inequities in MMR had also been reported in previous studies [4.7]. Besides the similar findings on regional inequities, our findings added to the evidence from the perspective of health services delivery. Gao and colleagues [7] used national data between 1997 to 2014 and found that the MMR in the western region was 118% higher than that in the eastern region (2.18, 1.44–3.28), and MMR in the central region was 41% higher than that in the eastern region (1.41, 0.99–2.01). Our results on regional inequities were slightly lower than those reported by Gao and colleagues [7], which might be related to the different scope of years in the two studies, and the regional differences in the early years are more pronounced. The regional inequities in MMR were gradually decreasing [4]. Another reason might be the different confounding factors controlled in these studies. We additionally controlled maternal healthcare factors which might be associated with maternal mortality, such as hospital delivery rates. It was worth noting that, we found MMR in the central region is fluctuating, unlike the continuous decline of MMR in the eastern and western regions. Our findings indicated that maternal health in the central region was not a major focus during the two-child policy era, which has not been reported in previous studies. One possible explanation of the findings is that the proportion of high-risk pregnant women in central China was significantly higher than that of the less economically-developed western region during the two-child policy era, while health resources were not as advanced as the better economically-developed eastern region. The supply of medical services could not meet the rapidly increased needs of high-level maternal and child healthcare in the central region, there is much pressure on preventing maternal deaths. The supplementary allocation of medical resources has been more invested in the western regions where medical resources have been limited in the past decades. How to strengthen the prevention of maternal mortality in central China is an important issue. There are some steps that can be taken in the future, for example, strengthening the case analysis of maternal deaths, optimizing the management process for the problems found at the service providing level, giving adequate consideration to the central region when building a new national or provincial referral center for critical illness of pregnant women.

In this study, we also found that the socio-demographic factors such as high proportion of ethnic minorities, inadequate length of highways, and low GDP per capita were related to high MMR, which was consistent with previous studies [7, 29, 30]. The association between high hospital delivery rates and low MMR was well established in previous studies [31]. Over the past two decades, financial subsidies for hospital deliveries for rural women largely increased the hospital delivery rate in the western regions, which promoted a rapid decline of MMR in the western regions [7]. At present, the national hospital delivery rate in the country has reached 99.8% [15]. However, since 2018, the Chinese government no longer allocates special funds to subsidize hospital deliveries for rural women. Medical expenses of hospital deliveries for rural women are only covered by basic medical insurance for urban and rural residents. The impact of the termination of financial subsidies for hospital deliveries on hospital delivery rates and MMR of women in the western poorest provinces needs to be assessed in the further studies.

Many countries still struggle to meet the SDGs target of fewer than 70 deaths per 100,000 livebirths by 2030 [28, 32]. Although the burden of MMR disproportionately occurs in low and middle-income countries (LMICs), it also affects increasing number of women in some high-income countries due to the increase in maternal deaths due to indirect causes in recent years [27, 28]. As one of the LMICs, China has the largest population in the world and has achieved remarkable progress in reducing MMR during MDGs period [4]. China's efforts on risk management to reduce MMR could provide experience for other countries. However, this study also has several limitations. First, this study is a provincial-based analysis based on national data, and has not been analyzed at the county level. However, at the county level, there will be of greater fluctuation of MMR due to the relatively small number of livebirths and the smaller number of observed maternal deaths in individual counties, while provincial level data can avoid this situation. Second, due to the lack of reliable data on the characteristics of pregnant women in different provinces (such as the proportion of advanced maternal age, prevalence of gestational hypertension and scarred uterus), we did not include the characteristics of pregnant women in multivariate analysis, which might have an impact on the further analysis of the results. Third, the long-term effect after the implementation of the risk management strategy cannot be evaluated in this study. It is necessary to further evaluate the long-term effects of the risk management strategy in the future. Fourth, we used the MMR data from the National Health Statistics Yearbooks, that the underestimation of MMR different from the national individual-level data might lead to the underestimation on the effect of the FSMNS risk management package. Fifth, the data in this study up to 2017 that the impact of COVID-19 on MMR could not be examined in this study. Some studies had reported the increase of MMR and other adverse outcomes caused by the COVID-19 pandemic [33, 34]. The optimal strategy on risk management of infectious disease and guarantee of maternal health services during the pandemic to reduce the impact on maternal health needs to be explore in the future.

In conclusion, the national risk management strategy among pregnant women contributed to the stable decline of MMR in China during the two-child policy era. China's efforts on risk management to reduce MMR could also provide experience for other countries. More attention should be paid to pregnant women in central China with obviously fluctuant MMR in recent years, and in western China with higher MMR but limited health resources. China should continue to implement risk management based on FSMNS, strengthen the screening of high-risk groups and the training of maternal and child health workers in the future, to ensure reaching the targets of SDGs and the Healthy China Plan by 2030 on time.

## Availability of data and materials section

The datasets generated and analyzed during the current study are available in the database of yearbooks in the CNKI platform (https://data.cnki.net/area/yearbook/search?v=&iss=0&t=c&z=D09).

## Supplementary Information

Below is the link to the electronic supplementary material.

**Additional file 1:** **Supplemental Figure 1.** Maternal mortality ratio and its annualised rate of decline by province, 2008-2017. (A) Maternal mortality ratio by province in 2017. (B) Annualised rate of decline in maternal mortality ratio by province, 2008-2017. **Supplemental Box 1.** **Supplemental table 1.** Association between the proportion of pregnant women at high risk and maternal mortality stratified by the implementation of risk management.
