# Supplementary material for: Risk management of pregnant women and the associated low maternal mortality from 2008–2017 in China: a national longitude study
Source: BMC Health Serv Res. 2022 Mar 14;22:335. doi: 10.1186/s12913-022-07721-z (PMC8920427; doi:10.1186/s12913-022-07721-z)
Supplement: Supplementary file 1 — Additional file 1: Supplemental Figure 1. Maternal mortality ratio and its annualised rate of decline by province, 2008-2017. (A) Maternal mortality ratio by province in 2017. (B) Annualised rate of decline in maternal mortality ratio by province, 2008-2017. Supplemental Box 1. Supplemental table 1. Association between the proportion of pregnant women at high risk and maternal mortality stratified by the implementation of risk management. [file 12913_2022_7721_MOESM1_ESM.docx]

**Supplemental files**

**Supplemental Figure 1. Maternal mortality ratio and its annualised rate of decline by province, 2008-2017. (A) Maternal mortality ratio by province in 2017. (B) Annualised rate of decline in maternal mortality ratio by province, 2008-2017.**


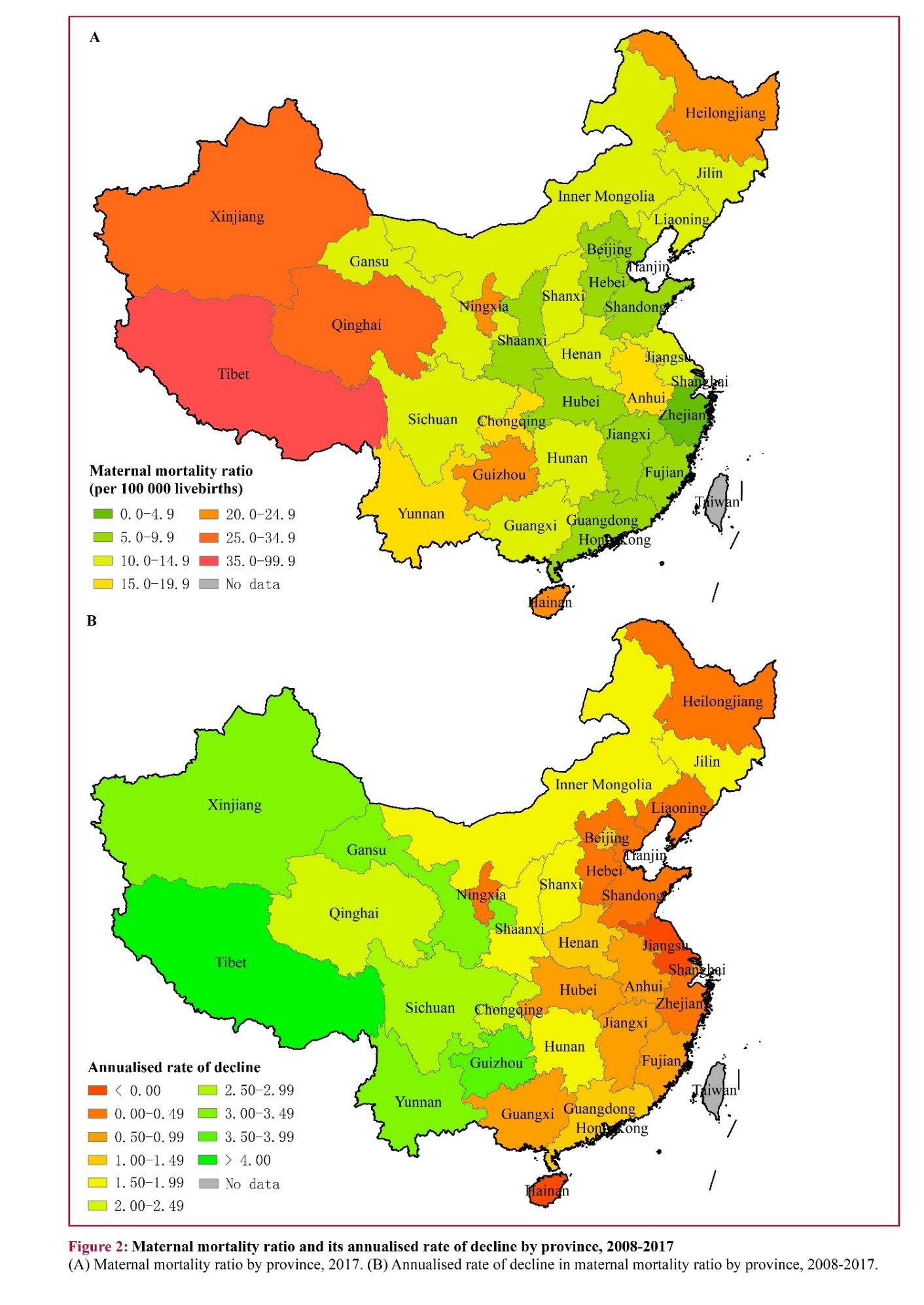


**Supplemental Box 1.**

**Box 1**

**Key points in the Five Strategies for Maternal and Newborn Safety**

- **Pregnancy risk screening and assessment**

Pregnancy risk screening for pregnant women is carried out during the first visit to a healthcare facility. The secondary or tertiary healthcare facilities utilize midwifery services to conduct pregnancy risk assessments and classify pregnancy risk according to risk severity. Medical records are labelled with five colors of “green (low risk), yellow (general risk), orange (high risk), red (highest risk), and purple (infectious disease) to enhance classification management. The yellow, orange, red, and purple labelled pregnant women are recommended to receive maternal health services and hospital delivery at secondary or tertiary health facilities.

- **Case-by-case management of high-risk pregnancies**

Pregnant women with different risk levels are transferred to different levels of MCH institutions for perinatal care and delivery (Figure 9). High risk mothers (labelled with orange, red, and purple) are included for the case-by-case management as the key population during the perinatal period to ensure whole-process management, dynamic supervision, centralized treatment, and to ensure the screening, registration, reporting, management, and treatment of every high-risk pregnant woman.

- **Critically ill pregnant women and newborn referral and treatment**

A nationwide network of critically ill pregnant women and newborn referral and treatment has been established, including a total of 3 369 maternal critical care centers and 3 070 neonatal critical care centers at the province, city, and county levels. A multidisciplinary maternal and neonatal critical care expert group has been established in each region. Maternal and neonatal critical care centers are responsible for critical care in designated areas, and they serve to keep the green channel open for critical illness referrals.

- **Maternal death case reporting**

Statistical tables of orange and red labelled pregnant women have been established to guide the effective management and treatment of high-risk pregnant women. China launched a direct reporting system for individual maternal deaths for the first time on October 1, 2017. Within two hours of a maternal death, health facilities should report the case to the designated county-level maternal and child health care institutions. After checking the situation, designated personnel located at the county-level maternal and child healthcare institution should report detailed information of the death case using the national annual online reporting information system for maternal and child health before the 10th day of each month. After a review of maternal deaths, rectification measures should be implemented by related health facilities.

- **Accountability**

Areas that have achieved outstanding progress in the reduction of MMR should summarize and promote of these effective experiences in a timely manner and be praised by the National Health Commission. For areas with increasing MMR trends, a group of experts are assigned in time to give trainings to a targeted guidance. For the areas that have not implemented the required measures to reduce MMR, the National Health Commission will issue a criticism by circulating a notice. If a hospital occurs maternal deaths continuously, the director of the related hospital will be required to be accountable.

| **Supplemental table 1. Association between the proportion of pregnant women at high risk and maternal mortality stratified by the implementation of risk management** | | | | | |
| --- | --- | --- | --- | --- | --- |
| Factors | Before risk management (2008-2013) | |  | After risk management (2014-2017) | |
|  | Adjusted risk ratio (95% CI) | P Value |  | Adjusted risk ratio (95% CI) | P Value |
| Proportion of pregnant women at high risk (%) | 0.99 (0.98,1.00) | 0.183 |  | 0.99 (0.98,1.00) | 0.014* |
| Region |  |  |  |  |  |
| Eastern | 1 (Reference) | - |  | 1 (Reference) | - |
| Central | 1.28 (1.05,1.57) | 0.017* |  | 1.26 (1.04,1.52) | 0.016* |
| Western | 1.34 (1.07,1.69) | 0.012* |  | 1.41 (1.10,1.81) | 0.007* |
| Year | 0.96 (0.94,0.99) | 0.003* |  | 1.03 (0.97,1.09) | 0.333 |
| Proportion of female illiterates aged 15 years or over (%) | 1.01 (1.00,1.02) | 0.034* |  | 1.01 (0.99,1.02) | 0.410 |
| Proportion of ethnic minorities (%) | 1.01 (1.01,1.02) | <0.001* |  | 1.02 (1.01,1.02) | <0.001* |
| Length of highways (1000 km) | 0.95 (0.90,1.00) | 0.044* |  | 0.95 (0.90,0.99) | 0.017* |
| Crude birth rates (%) | 0.97 (0.93,1.00) | 0.081 |  | 0.98 (0.95,1.01) | 0.258 |
| GDP per capita (10 000 RMB) | 0.86 (0.80,0.92) | <0.001* |  | 0.97 (0.91,1.04) | 0.424 |
| Number of licensed doctors and nurses per 1000 population | 1.05 (0.98,1.12) | 0.137 |  | 1.02 (0.90,1.15) | 0.736 |
| Number of beds of gynaecology, obstetrics, and paediatrics  per 1000 livebirths | 1.00 (0.99,1.00) | 0.456 |  | 1.00 (1.00,1.00) | 0.986 |
| Government health expenditures per capita (1000 RMB) | 1.51 (1.20,1.92) | 0.001* |  | 0.91 (0.69,1.20) | 0.495 |
| Proportion of maternal systematic management (%) | 1.00 (0.99,1.01) | 0.990 |  | 1.00 (1.00,1.01) | 0.244 |
| Hospital delivery rate (%) | 0.99 (0.98,1.00) | 0.142 |  | 1.02 (0.97,1.08) | 0.346 |
| Proportion of skilled birth attendance and sterile delivery (%) | 1.01 (1.00,1.01) | 0.065 |  | 0.82 (0.51,1.31) | 0.400 |
| *P<0.05. Risk ratios were adjusted for sociodemographic factors, health resource, and other maternal health factors. | | | | | |

**Supplemental file 1. the interview questionnaire**

1. **Year**

- **2008 □ 2009 □ 2010 □ 2011 □ 2012 □ 2013**
- **2014 □ 2015 □ 2016 □ 2017**

1. **Province:**
2. **Maternal mortality ratio: per 100,000 livebirths**
3. **The number of livebirths:**
4. **Proportion of pregnant women at high risk: (%)**
5. **Proportion of maternal systematic management: (%)**
6. **Hospital delivery rate: (%)**
7. **Proportion of skilled birth attendance and sterile delivery: (%)**
8. **Government health expenditures per capita: (RMB)**
9. **The number of licensed doctors per 1000 population:**
10. **The number of licensed nurses per 1000 population:**
11. **The number of beds in medical institutions per 1000 population:**
12. **The number of beds of gynaecology, obstetrics, and paediatrics in medical institutions:**
13. **The number of resident populations:**
14. **Proportion of female illiterates aged 15 years or over: (%)**
15. **Proportion of ethnic minorities: (%)**
16. **Length of highways:**
17. **Crude birth rates:**
18. **Gross domestic product (GDP) per capita:**
19. **The number of populations:**
20. **The number of livebirths:**
